# Effects of feeding diets without mineral P supplement on intestinal phytate degradation, blood concentrations of Ca and P, and excretion of Ca and P in two laying hen strains before and after onset of laying activity

**DOI:** 10.1016/j.psj.2024.104407

**Published:** 2024-10-10

**Authors:** Vera Sommerfeld, Jörn Bennewitz, Amélia Camarinha-Silva, Martina Feger, Michael Föller, Korinna Huber, Michael Oster, Siriluck Ponsuksili, Sonja Schmucker, Jana Seifert, Volker Stefanski, Klaus Wimmers, Markus Rodehutscord

**Affiliations:** ⁎ Institute of Animal Science, University of Hohenheim, Stuttgart, 70599, Germany; † Department of Physiology, University of Hohenheim, Stuttgart, 70599, Germany; ‡ Research Institute for Farm Animal Biology (FBN), Dummerstorf, 18196, Germany

**Keywords:** gene expression, laying hen, *myo*-inositol, phosphorus, phytate

## Abstract

The objective of this study was to characterize intestinal phytate degradation and mineral utilization by 2 laying hen strains before and after the onset of egg laying using diets without or with a mineral phosphorus (**P**) supplement. One offspring of 10 roosters per strain (Lohmann Brown-classic [**LB**] and Lohmann LSL-classic [**LSL**]) was sacrificed before (wk 19) and after (wk 24) the onset of egg-laying activity and following 4 wk placement in a metabolic unit. Diets were corn-soybean meal-based and without supplemented P (**P-**) or with 1 g/kg supplemented P (**P+**) from monocalcium phosphate. In wk 19 and 24, the blood plasma and digesta of duodenum+jejunum and distal ileum were collected. The concentration of P in blood plasma was higher in hens fed P+ than P- (*P* < 0.001). In duodenum + jejunum and ileum content, the concentrations of InsP_6_, Ins(1,2,4,5,6)P_5_ and Ins(1,2,3,4,5)P_5_ were lower in P- than in P+ (*P* ≤ 0.009). In duodenum+jejunum, the concentrations of InsP_6_, Ins(1,2,4,5,6)P_5_ and Ins(1,2,3,4,5)P_5_ were lower in wk 24 than 19 and lower in LSL than LB hens (*P* < 0.001). The concentration of *myo*-inositol (**MI**) in duodenum + jejunum content was lower in wk 19 than 24 (*P* < 0.001). Following a 4-d total excreta collection, the retained amount of P was higher in P+ than P- (*P* < 0.001). Phosphorus retention was lower in LB hens fed P- than in other treatments (P × strain: *P* = 0.039). In the jejunal tissue, some genes related to intracellular InsP metabolism were higher expressed in LB than LSL hens. The renunciation of mineral P increased endogenous phytate degradation, but more P was retained with supplemented P. Differences in endogenous phytate degradation between the periods before and after the onset of egg laying might be attributed to different Ca concentrations in intestinal digesta caused by different Ca needs in both periods.

## INTRODUCTION

Laying hens rely on a continuous supply of phosphorus (**P**). For this reason, commercial laying hen feed is commonly supplemented with P from mineral sources, which can have environmental, economic, and resource-expending consequences. The amount of P in a laying hen feed originating from common plant ingredients would be adequate if the plant-P was completely available for laying hens. However, the majority of P in plant ingredients is bound as phytate, the salt of *myo*-inositol 1,2,3,4,5,6-hexakis phosphate (**InsP_6_**). Phytate needs phytases and other phosphatases to release its phosphate groups before they become available for absorption in the intestine. As non-ruminants have only low endogenous phytase activity, the digestibility of phytate-P is low if no enzyme is supplemented. However, a reduction of mineral P supplementation in the feed can increase endogenous phytate degradation and thus phytate-P digestibility in broiler chicken (Rodehutscord et al., 2022). This may be of interest to laying hen feeding because an increasing number of studies suggested that the P requirement of laying hens is lower than currently recommended (Ahmadi and Rodehutscord, 2012; Rodehutscord et al., 2023). Alongside phytate degradation, dietary P reduction can increase *myo*-inositol (**MI**) release in the intestinal tract, the second degradation end product. *Myo*-inositol itself is known for its many cellular functions and involvement in cell structure (Huber, 2016).

A reduction of dietary non-phytate-P (**NPP**) concentration by 20% (from 2.5 g/kg) was without major effects on performance and physiological traits in the 2 high-performing laying hen strains Lohmann Brown-Classic (**LB**) and Lohmann LSL-Classic (**LSL**) (Sommerfeld et al., 2020b). However, LB hens had higher endogenous phosphatase activity, concentrations of InsP isomers in the small intestine, and MI concentrations in blood and eggs than LSL hens (Sommerfeld et al., 2020a,b). The LB hens also showed higher mRNA expression of 2 sodium/phosphate cotransporters in the ileum than LSL hens (Sommerfeld et al., 2020b) and differences between the strains were found for blood hormones related to P and calcium (**Ca**) homeostasis, such as calcidiol, calcitriol, estradiol, and triiodothyronine, partly affected by the production period of the hens (Omotoso et al., 2021; Reyer et al., 2021). Thus, besides the genetic strain, the age or stage of production of the hens is relevant for P metabolism. Concentrations of plasma P and P utilization were higher in wk 16 than wk 24 of age, and vice versa for plasma Ca and ileal InsP_6_ and Ca (Sommerfeld et al. 2020a). Blood concentrations of parathyroid hormone, triiodothyronine, estradiol, vitamin D forms, Ca, and several plasma metabolites also were markedly different between hens before and after the onset of egg-laying (Gonzalez-Uarquin et al., 2021; Omotoso et al., 2021). However, it could not be distinguished whether these differences were due to the changing metabolism in these production periods (before and after the onset of laying activity) or due to the diets that had been adapted in their composition to the requirements of the respective production period.

The objective of the present study was to expose hens from 2 different genetic strains to a diet without any mineral P supplementation before and after the onset of egg-laying activity. We hypothesized that 1) the renunciation of mineral P provokes mechanisms that increase endogenous phytate degradation and P retention in laying hens, 2) the 2 laying hen strains react differently to the dietary P reduction, 3) differences in metabolic responses exist between hens in wk 19 and wk 24 of age, and 4) a higher phytate degradation due to low mineral P leads to shifts in intestinal digesta composition which are reflected by components of intracellular InsP signaling in the proximal intestine.

## MATERIALS AND METHODS

This study was part of the interdisciplinary Research Unit P-Fowl – Inositol phosphates and *myo*-inositol in the domestic fowl: Exploring the interface of genetics, physiology, microbiome, and nutrition (https://p-fowl.uni-hohenheim.de/). The animal trial was conducted at the Agricultural Experiment Station of the University of Hohenheim, Germany. It was approved by the Regierungspräsidium Tübingen, Germany (Project no. HOH67-21TE) and conducted in accordance with German animal welfare legislation. The experiment was designed as a 2 × 2 × 2 factorial arrangement of treatments with 2 laying hen strains (LB and LSL), 2 hen production periods (before and after the onset of egg-laying), and 2 levels of mineral P supplement (without supplemented P (**P-**) or with 1 g/kg supplemented P (**P+**) from monocalcium phosphate).

### Birds and Housing

A total of 240 LB and 240 LSL female chicken hatchlings were obtained from the breeding company (Lohmann Breeders GmbH, Cuxhaven, Germany), representing 2 distinct genetic backgrounds. For each strain, 12 nonrelated roosters were used for mating. Twenty offspring per rooster were taken and placed at the beginning of the study. Birds were raised in floor pens on deep litter bedding according to the standardized routine procedure of the station, including the vaccination program.

**Figure 1:**
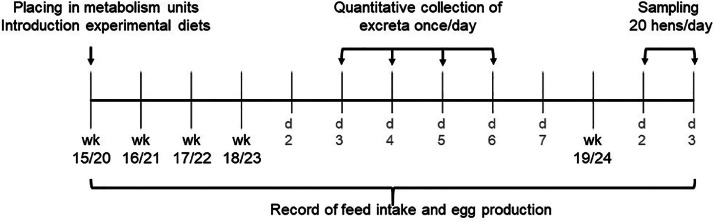
Timeline of the experimental procedures.

The procedures in the experimental periods are displayed in Figure 1. At 2 time periods, hens used for the experiment spent 4 weeks individually in metabolic units. At the end of these periods, total excreta were collected for 4 d, followed by sacrificing and sampling of digesta, organs, and tissues of the hens. At the beginning of each period, either immediately before (wk 15) or after (wk 20) the onset of egg-laying activity, selected hens were moved to the metabolic units. Before placement, the offspring of 10 out of the 12 roosters per strain were chosen based on the average BW. In both wk, one randomly chosen offspring of each of the 10 roosters per strain was placed in one metabolic unit (1 m × 1 m × 1 m), resulting in 10 replicates per strain, diet and wk, in a randomized complete block design. Metabolic units were equipped with a wooden perch, a nest, a feeding trough, water cups, and a wire mesh floor. For total excreta collection, stainless-steel trays were placed underneath the units. Nine hours of light and 15 h of darkness were provided during the first period, then gradually changed to obtain 16 h of light and 8 h of darkness at the end of the second period. The temperature in the barn was set to 18 to 22°C during the periods.

### Diets

The experimental diets were based on corn and soybean meal to minimize plant intrinsic phytase activity (Table 1). Diets were calculated to contain adequate levels of all nutrients according to the recommendations of the Gesellschaft für Ernährungsphysiologie (**GfE**) (Gesellschaft für Ernährungsphysiologie, 1999), except for P, contained TiO_2_ as an indigestible marker and were provided in mash form. Exogenous phytase was not added. Diets were mixed in the certified feed mill of the Agricultural Experiment Station of the University of Hohenheim. Feed and water were provided for ad libitum consumption. The calculated nutrient concentrations were confirmed by analyses overall (Table 2).

**Table 1:** Ingredient and calculated composition of the experimental diets.

	Developer		Prelayer		Layer	
*Ingredients, g/kg*	P-	P+	P-	P+	P-	P+
Corn	619.5	617.1	589.8	585.8	599.2	595.9
Soybean meal	220.0	220.0	275.5	276.2	260.0	260.0
Alfalfa meal	120.0	120.0	60.0	60.0	30.0	30.0
Soybean oil	8.0	8.0	10.0	10.0	15.0	15.0
DL-Methionine	1.5	1.5	3.7	3.7	4.5	4.5
L-Lysine sulphate	-	-	-	-	0.7	0.7
Monocalcium phosphate	-	4.7	-	5.0	-	5.0
Limestone, fine	18.2	15.9	27.5	26.8	23.1	22.4
Limestone, coarse	-	-	20.0	19.0	54.0	53.0
Sodium chloride	2.3	2.3	2.8	2.8	3.0	3.0
Choline chloride	1.0	1.0	1.0	1.0	1.0	1.0
Sodium bicarbonate	2.0	2.0	2.2	2.2	2.0	2.0
Vitamin mix^1^	2.0	2.0	2.0	2.0	2.0	2.0
Mineral mix^2^	0.5	0.5	0.5	0.5	0.5	0.5
TiO_2_	5.0	5.0	5.0	5.0	5.0	5.0
*Calculated, g/kg*						
P	3.0	4.0	3.3	4.3	3.1	4.1
Non phytate-P	1.3	2.3	1.4	2.4	1.3	2.3
Ca	9.0	9.0	22.5	22.5	35.0	35.0
CP	155	155	176	176	168	168
ME, MJ/kg	11.6	11.6	11.5	11.5	11.5	11.5

The hens used in the first period received a developer feed the first 10 d after placement in the metabolic units at wk 15, a prelayer feed the next 7 d, and a layer feed from d 17 until the end of the period, each with or without mineral P supplementation. The hens of the second period received the layer feed from the d of placement into the metabolism units at wk 20 until the end.

1 Vitamin premix (Miavit GmbH, Essen, Germany), provided per kg of the complete diet: 10,000 IU vitamin A, 3,000 IU vitamin D3, 30 mg vitamin E, 2.4 mg vitamin K3, 100 mcg biotin, 1 mg folic acid, 3 mg vitamin B1, 6 mg vitamin B2, 6 mg vitamin B6, 30 mcg vitamin B12, 50 mg nicotinamide, 14 mg calcium-D-pantothenate.

2 Trace element premix (Gelamin Gesellschaft für Tierernährung mbH, Memmingen, Germany), provided per kg of complete diet: 80 mg manganese from manganese-(II)-oxide, 60 mg zinc from zinc sulfate monohydrate, 25 mg iron from ferrous-(II)-sulfate monohydrate, 7.5 mg copper from cupric-(II)-sulfate pentahydrate, 0.6 mg iodine from calcium iodate, 0.2 mg selenium from sodium selenite.

**Table 2:** Analyzed composition of the experimental layer diets.

*g/kg DM*	P-	P+
P	3.6	4.9
Ca	38.2	35.7
CP	187	187
*myo*-inositol	0.4	0.4
Ins(1,2,4,5,6)P_5_	0.3	0.3
InsP_6_	6.2	6.3
InsP_6_-P	1.8	1.8
NPP	1.8	3.1
Ti	3.3	3.2
*µmol/g DM*^1^		
*myo*-inositol	2.0	2.0
Ins(1,2,3,4,5)P_5_	<LOQ	<LOQ
Ins(1,2,4,5,6)P_5_	0.6	0.6
InsP_6_	9.5	9.5

1 All other InsP isomers below the limit of quantification (LOQ) or limit of detection.

### Experimental Procedures, Samplings, and Measurements

The hens used in the first period received a developer feed the first 10 d after placement in the metabolic units at wk 15, a prelayer feed the next 7 d, and a layer feed from d 17 until the end of the period, each with or without mineral P supplementation. The hens of the second period received the layer feed from the same batch from the day of placement in the metabolism units at wk 20 until the end. The animals were inspected at least twice daily. The eggs of all hens were collected and weighed. One egg per hen was taken during the 4 excreta collection days and kept at room temperature until further processing. Feed and individual hens were weighed at the beginning and the end of each period and excreta collection. Total excreta were collected from the trays at 24-h intervals for 4 consecutive days in the morning. Feathers, skin scales, and spilled feed were carefully removed from the trays before every collection and the excreta were immediately frozen at -20°C. Feed leftovers from the trays and troughs were weighed, and feed intake was corrected for these values.

Before slaughtering in wk 19 and 24, the feed was deprived for 1 h, followed by 1 h ad libitum access to the feed to standardize gut fill. The hens were individually stunned with a gas mixture of 35% CO_2_, 35% N_2_, and 30% O_2_ and killed by bleeding. Trunk blood was collected in tubes containing sodium fluoride for MI analysis or lithium heparin for P and Ca analysis. Blood samples were centrifuged for 10 min at 2,500 × *g* to obtain the plasma. Digesta from the duodenum and jejunum (**duodenum+jejunum**) together, and the terminal part of the ileum, defined as the posterior two-thirds of the section between Meckel´s diverticulum and 2 cm prior to the ileo-ceco-colonic junction, were collected by gentle squeezing. Digesta samples were immediately frozen at -20°C, freeze-dried, and pulverized (PULVERISETTE 9, Fritsch GmbH, Idar-Oberstein, Germany). Pulverized samples were stored in airtight containers until further analysis.

Yolk and albumen from one egg per hen were separated and weighed. Then, they were freeze-dried, weighed again, pulverized with pestle and mortar, and stored until further analysis.

### Sample Preparation and Analyses

Excreta samples were thawed at +3°C, weighed, pooled for each hen in each period, and homogenized. Excreta DM was analyzed in triplicate. A subsample of the excreta was freeze-dried, pulverized, and stored as described for digesta. Feed was ground to pass through a 0.5 mm sieve (Ultra Centrifugal Mill ZM 200, Retsch GmbH, Haan, Germany). Samples were analyzed for DM according to the official method in Germany (method no. 3.1) (Verband Deutscher Landwirtschaftlicher Untersuchungs- und Forschungsanstalten (VDLUFA), 2007).

Pulverized feed, digesta, and excreta samples were analyzed for P, Ca, and titanium (**Ti**) by inductively coupled plasma-optical emission spectrometry following wet digestion using a modified method of Boguhn et al. (2009), described in detail by Zeller et al. (2015). Extraction and measurement of InsP_3-6_ isomers in feed and digesta were carried out using the method of Zeller et al. (2015) with modifications by Sommerfeld et al. (2018) and high-performance ion chromatography detection (ICS-3000 system, Dionex, Idstein, Germany). This methodology does not allow to distinguish between the D- and L-forms of the isomers. *Myo*-inositol concentrations in feed, digesta, yolk, albumen, and plasma were analyzed using a gas chromatograph/mass spectrometer following derivatization of the samples (Sommerfeld et al., 2018). Plasma Ca was measured by the Arsenazo method and inorganic P (**P_i_**) as phosphomolybdate in a Beckman Olympus AU480 photometer by IDEXX BioResearch Vet Med Labor GmbH (Ludwigsburg, Germany).

Jejunum mucosa samples were collected, immediately placed on dry ice, and stored at -80°C until RNA extraction. Total RNA was extracted from approximately 50 mg of each sample using TRIzol Reagent (Invitrogen) followed by purification with the RNeasy Mini kit (Qiagen) including DNaseI treatment. RNA quality was assessed using an Agilent 2100 Bioanalyzer. For mRNA analysis, 200 ng of mRNA was reverse transcribed using the Reverse Transcription Master Mix (Fluidigm PN 100-6297). The resulting cDNA was used for qPCR, specifically targeting 16 transcripts related to InsP metabolism pathways, performed on the Fluidigm BioMark HD System. Specific target amplification (**STA**) was conducted using the PreAmp Master Mix (Fluidigm PN 1005581) according to the manufacturer's recommendations. Pre-amplification sample mixtures were prepared using the PreAmp Master Mix (Fluidigm PN 1005581). Fluidigm quantitative measurement runs were carried out with 96.96 dynamic arrays (Fluidigm Corporation, CA) according to manufactures instructions. Data were analyzed using real-time PCR software on the BioMark HD instrument, with normalization to housekeeping genes (*ACTB, TBP, RPL13*, and *GAPDH*).

### Calculations and Statistical Analysis

Calcium and P retention was calculated as the proportion of intake that was not recovered in excreta based on quantitative data.

The comparisons of treatments were performed using the MIXED procedure and pairwise t-tests using the software package SAS (version 9.3; SAS Institute Inc., Cary, NC). The individual hen was considered as the experimental unit. The following model was used:

$$Y_{\text{ijklm}}\;=\;\mu \;+\;\alpha_{i}\;+\beta_{j}\;+\;\gamma_{k}\;+\;{\left(\alpha \beta \right)}_{\text{ij}}\;+\;{\left(\alpha \gamma \right)}_{\text{ik}}\;+\;{\left(\text{βγ}\right)}_{\text{jk}}\;+\;{\left(\alpha \beta \gamma \right)}_{\text{ijk}}\;+\;\delta_{l}\;+\phi_{m}\;+\;\varepsilon_{\text{ijklm}},$$

where Y_ijklm_ = response variable, μ = overall mean, α_i_ = effect of strain (fixed), β_j_ = effect of hen production period (fixed), γ_k_ = effect of mineral P supplementation, all 2- and 3-fold-interactions among strain, production period and mineral P supplementation (fixed), δ_l_ = block (random), ϕ_m_ = father/rooster (random), and ε_ijklm_ = residual error. Statistical significance was declared at *P* < 0.05.

## RESULTS

Body weight gain and ADFI of hens measured over the 4 wk in the metabolic units were higher in wk 24 than 19 (Table 3; *P* < 0.001). Body weight gain and egg weight were higher for LB than LSL hens (*P* < 0.01).

**Table 3:** Effect of production period, dietary P level and hen strain on body weight (BW) gain, average daily feed intake (ADFI), egg number, and egg weight of laying hens.

			BW gain^1^	ADFI^1^	Egg number^2^	Egg weight^2^	BW^3^	ADFI^4^	Egg weight^4^
Period	P level	Strain	g	g/d		g	g	g/d	g
Wk 19	P+^5^	LB^7^	181	51	-	-	1361	59	-
Wk 19	P+	LSL^8^	88	45	-	-	1119	48	-
Wk 19	P-6	LB	146	49	-	-	1348	56	-
Wk 19	P-	LSL	89	43	-	-	1092	47	-
Wk 24	P+	LB	297	77	9	54	1790	97	54
Wk 24	P+	LSL	227	78	12	50	1484	97	51
Wk 24	P-	LB	268	80	11	52	1785	100	56
Wk 24	P-	LSL	231	73	12	48	1443	98	50
	pooled SEM		18.0	2.7	1.5	1.0	31.2	3.6	1.2
*P* values	Period		< 0.001	< 0.001	-	-	< 0.001	< 0.001	.
	Strain		< 0.001	0.082	0.182	0.003	< 0.001	0.090	< 0.001
	P		0.208	0.460	0.631	0.056	0.331	0.991	0.920
	P × Strain		0.146	0.203	0.342	0.905	0.570	0.991	0.238
	Period × strain		0.367	0.314	-	-	0.093	0.086	.
	Period × P		0.856	0.684	-	-	0.951	0.349	.
	Period × P × strain		0.974	0.224	-	-	0.802	0.787	.

Data are given as LSmeans; n = 10 hens.

1 In the whole period (4 weeks) until the respective day of slaughter.

2 Until the day before slaughter.

3 At the end of the excreta sampling.

4 During 4 d excreta sampling.

5 Supplemented with 1 g P/kg feed.

6 Without mineral P supplementation.

7 LB = Lohmann Brown-Classic.

8 LSL = Lohmann LSL-Classic.

The concentration of P_i_ in blood plasma was higher in hens fed P+ than in hens fed P- (Table 4; *P* < 0.001). Plasma Ca concentration was overall higher in wk 24 than in wk 19 and lower in hens fed P+ than in hens fed P- in wk 19 and vice versa in wk 24 (period × P: *P* = 0.003). The concentration of MI in blood plasma was higher in hens fed P- in wk 24 than in all other treatments (period × P: *P* = 0.003) and higher in LB fed P- than in LB fed P+ and LSL fed P- (P × strain: *P* = 0.009). The concentration of MI in egg albumen was lower for P+ than P- (Table 4; *P* = 0.02), whereas the concentration of MI in egg yolk was not affected by the treatments.

**Table 4:** Effect of production period, dietary P level, and hen strain on plasma traits and *myo*-inositol (MI) in the egg of laying hens.

			Plasma P	Plasma Ca	Plasma MI	Albumen MI	Yolk MI
Period	P level	Strain	mmol/L	mmol/L	mmol/L	µmol/g DM	µmol/g DM
Wk 19	P+^1^	LB^3^	1.9	2.8	0.12	-	-
Wk 19	P+	LSL^4^	1.8	3.0	0.13	-	-
Wk 19	P-2	LB	1.4	3.3	0.12	-	-
Wk 19	P-	LSL	1.2	3.7	0.10	-	-
Wk 24	P+	LB	1.8	7.1	0.12	3.6	1.8
Wk 24	P+	LSL	1.7	6.8	0.13	4.2	1.8
Wk 24	P-	LB	1.4	6.3	0.16	4.4	2.0
Wk 24	P-	LSL	1.3	6.4	0.14	4.6	1.7
	pooled SEM		0.08	0.27	0.001	0.24	0.07
Wk 19	P+			2.9^d^	0.12^b^		
Wk 19	P-			3.5^c^	0.11^b^		
Wk 24	P+			6.9^a^	0.12^b^		
Wk 24	P-			6.4^b^	0.15^a^		
	P+	LB			0.12^b^		
	P+	LSL			0.13^ab^		
	P-	LB			0.14^a^		
	P-	LSL			0.12^b^		
*P* values	Period		0.228	< 0.001	0.001	.	.
	Strain		0.055	0.667	0.545	0.079	0.160
	P		< 0.001	0.926	0.179	0.020	0.110
	P × Strain		0.641	0.364	0.009	0.294	0.078
	Period × Strain		0.709	0.243	0.963	.	.
	Period × P		0.165	0.003	0.003	.	.
	Period × P × Strain		0.709	0.702	0.744	.	.

Data are given as LSmeans; n = 10 hens.

1 Supplemented with 1 g P/kg feed.

2 Without mineral P supplementation.

3 LB = Lohmann Brown-Classic.

4 LSL = Lohmann LSL-Classic.

a–d Different superscript letters within a column and means of two-way interactions indicate significant interaction effects among two factors (*P* < 0.05).

In duodenum+jejunum content, the concentrations of InsP_6_, Ins(1,2,4,5,6)P_5_, and Ins(1,2,3,4,5)P_5_ were lower in wk 24 than 19, lower in LSL than LB hens, and lower in hens fed P- than in hens fed P+ (Table 5; *P* ≤ 0.001). The concentration of MI in duodenum+jejunum content was lower in wk 19 than 24 (*P* < 0.001). Calcium concentration in duodenum+jejunum content was highest in hens fed P+ in wk 19, lowest in hens fed P+ in wk 24 and in-between in hens fed P- at both periods (period × P: *P* = 0.04). The concentration of P in duodenum+jejunum was higher in LB hens than in LSL hens fed P+ in wk 19. All other treatments had lower concentrations but did not differ from each other (3-way interaction: *P* = 0.036).

**Table 5:** Effect of production period, dietary P level, and hen strain on concentrations of *myo*-inositol, InsP isomers, Ca, P, and Ti in the duodenum+jejunum content of laying hens at different production periods.

			*myo*-inositol	Ins(1,2,3,4,5)P_5_	Ins(1,2,4,5,6)P_5_	InsP_6_	Ca	P	Ti
Period	P level	Strain	µmol/g DM				g/kg DM		
Wk 19	P+^1^	LB^3^	5.6	0.9	1.8	23.4	62.6	12.0^a^	8.1
Wk 19	P+	LSL^4^	5.9	0.7	1.3	18.0	63.0	10.6^b^	6.2
Wk 19	P-2	LB	6.1	0.8	1.5	20.2	59.0	8.1^c^	6.7
Wk 19	P-	LSL	6.7	0.6	1.0	16.7	48.9	8.7^c^	5.6
Wk 24	P+	LB	7.4	0.6	1.1	18.5	42.4	8.2^c^	6.3
Wk 24	P+	LSL	7.7	0.4	0.6	15.6	36.1	7.9^c^	4.8
Wk 24	P-	LB	7.7	0.5	0.8	16.3	44.3	8.2^c^	6.0
Wk 24	P-	LSL	7.1	0.4	0.5	13.2	46.7	7.8^c^	4.6
	pooled SEM		0.37	0.05	0.09	0.96	5.20	0.36	0.40
									
Wk 19	P+						62.8^a^		
Wk 19	P-						53.9^ab^		
Wk 24	P+						39.2^c^		
Wk 24	P-						45.5^bc^		
*P* values	Period		< 0.001	< 0.001	< 0.001	< 0.001	< 0.001	< 0.001	< 0.001
	Strain		0.629	< 0.001	< 0.001	< 0.001	0.394	0.129	< 0.001
	P		0.365	0.001	< 0.001	0.001	0.720	< 0.001	0.021
	P × Strain		0.631	0.628	0.796	0.520	0.909	0.066	0.480
	Period × Strain		0.183	0.541	0.352	0.244	0.693	0.831	1.000
	Period × P		0.124	0.771	0.538	0.978	0.040	< 0.001	0.156
	Period × P × Strain		0.220	0.771	0.735	0.384	0.187	0.036	0.619

Data are given as LSmeans; n = 10 hens.

1 Supplemented with 1 g P/kg feed.

2 Without mineral P supplementation.

3 LB = Lohmann Brown-Classic.

4 LSL = Lohmann LSL-Classic.

a–c Different superscript letters within a column and means of 2- or 3-way interaction indicate significant interaction effects among the two or 3 factors (*P* < 0.05).

In the distal ileum content, concentrations of InsP_6_, Ins(1,2,4,5,6)P_5_, and Ins(1,2,3,4,5)P_5_ were lower in hens fed P- than in hens fed P+ (Table 6; *P* ≤ 0.009). The concentration of MI was higher in LB than LSL hens in wk 19 but did not differ between strains in wk 24 (period × strain, *P* = 0.02). Ileal Ca concentration was higher in wk 19 than 24 (*P* < 0.001). Ileal P concentration in LB hens was higher when fed P+ compared to P- in wk 19 but not in wk 24. In LSL hens, ileal P concentration did not differ in wk 19 but was higher when fed P+ compared to P- in wk 24 (3-way-interaction: *P* = 0.02).

**Table 6:** Effect of production period, dietary P level, and hen strain on concentrations of *myo*-inositol, InsP isomers, Ca, P, and Ti in the distal ileum content of laying hens.

			*myo*-inositol	Ins(1,2,3,4,5)P_5_	Ins(1,2,4,5,6)P_5_	InsP_6_	Ca	P	Ti
Period	P level	Strain	µmol/g DM				g/kg DM		
Wk 19	P+^1^	LB^3^	2.1	1.1	2.1	29.1	85.8	12.0^a^	9.2
Wk 19	P+	LSL^4^	1.1	0.9	1.7	25.0	82.9	10.2^b^	7.5
Wk 19	P-2	LB	1.9	1.0	1.7	24.6	80.1	8.0^cd^	8.0
Wk 19	P-	LSL	1.3	0.9	1.5	25.2	83.0	8.9^bc^	7.3
Wk 24	P+	LB	1.6	0.8	1.2	25.7	65.4	8.1^cd^	8.2
Wk 24	P+	LSL	1.6	1.0	1.3	30.3	36.1	9.8^b^	7.9
Wk 24	P-	LB	1.8	0.7	1.0	22.6	68.7	7.3^d^	7.8
Wk 24	P-	LSL	1.5	0.8	1.0	24.7	59.1	8.1^cd^	8.7
		SEM	0.21	0.07	0.12	1.77	9.00	0.57	0.63
Wk 19	LB		2.0^a^	1.0^a^		26.9^a^			
Wk 19	LSL		1.2^c^	0.9^a^		25.1^a^			
Wk 24	LB		1.7^ab^	0.8^b^		24.1^a^			
Wk 24	LSL		1.5^bc^	0.9^a^		27.5^a^			
*P* values	Period		1.000	< 0.001	< 0.001	0.890	< 0.001	< 0.001	0.732
	Strain		< 0.001	0.507	0.200	0.528	0.095	0.355	0.233
	P		0.556	0.007	0.002	0.009	0.373	< 0.001	0.538
	P × Strain		0.847	0.731	0.751	0.658	0.272	0.220	0.183
	Period × Strain		0.020	0.005	0.086	0.035	0.093	0.028	0.073
	Period × P		0.844	0.349	0.551	0.364	0.169	0.060	0.240
	Period × P × Strain		0.169	0.137	0.551	0.131	0.548	0.020	0.941

Data are given as LSmeans; n = 10 hens.

1 Supplemented with 1 g P/kg feed.

2 Without mineral P supplementation.

3 LB = Lohmann Brown-Classic.

4 LSL = Lohmann LSL-Classic.

a–d Different superscript letters within a column and means of two-way interaction indicate significant interaction effects among two factors (*P* < 0.05).

Daily intake of Ca was higher in wk 24 than in wk 19 (Table 7; *P* < 0.001). Daily excretion of Ca was higher in LB than LSL hens (*P* < 0.001). The percentage Ca retention and retained amount of Ca were higher in wk 24 than in wk 19 (*P* < 0.001). Percentage Ca retention was higher in LSL than LB hens (*P* = 0.006). Daily intake of P was higher in wk 24 than wk 19 and higher in P+ than P- (period × P: *P* = 0.010). P excretion was higher in LB than LSL hens (*P* = 0.020) and higher in wk 24 than wk 19 (*P* < 0.001). P retention was higher in P+ than P- (*P* < 0.001) and lower in LB hens fed P- than in the other treatments (P × strain: *P* = 0.039).

**Table 7:** Effect of production period, dietary P level, and hen strain on Ca and P retention of laying hens.

			Ca intake	Ca excretion	Ca retention	Ca retention	P intake	P excretion	P retention	P retention
Period	P level	Strain	g/d	g/d	g/d	%	mg/d	mg/d	mg/d	%
Wk 19	P+^1^	LB^3^	1.83	1.23	0.60	33	260	155	105	40
Wk 19	P+	LSL^4^	1.51	0.98	0.53	36	214	138	76	35
Wk 19	P-2	LB	1.92	1.30	0.62	32	182	151	30	17
Wk 19	P-	LSL	1.62	1.00	0.62	38	153	111	41	27
Wk 24	P+	LB	3.24	1.26	1.98	61	440	328	112	24
Wk 24	P+	LSL	3.21	0.96	2.25	70	436	311	126	29
Wk 24	P-	LB	3.45	1.34	2.12	59	326	330	-5	-4
Wk 24	P-	LSL	3.38	0.94	2.45	72	319	264	55	18
		SEM	0.121	0.087	0.136	3.5	13.4	19.3	18.5	5.6
	P+	LB								32^a^
	P+	LSL								32^a^
	P-	LB								6^b^
	P-	LSL								22^a^
Wk 19	P+						237^c^			
Wk 19	P-						167^d^			
Wk 24	P+						438^a^			
Wk 24	P-						322^b^			
*P* values	Period		< 0.001	0.968	< 0.001	< 0.001	< 0.001	< 0.001	0.469	< 0.001
	Strain		0.100	< 0.001	0.283	0.006	0.089	0.020	0.366	0.101
	P		0.063	0.546	0.216	0.781	< 0.001	0.171	< 0.001	< 0.001
	P × Strain		0.922	0.504	0.692	0.383	0.692	0.194	0.088	0.039
	Period × Strain		0.097	0.579	0.062	0.145	0.067	0.618	0.071	0.134
	Period × P		0.558	0.866	0.519	0.861	0.010	0.808	0.118	0.620
	Period × P × Strain		0.857	0.828	0.975	0.861	0.569	0.638	0.887	0.951

Data are given as LSmeans; n = 10 hens.

1 Supplemented with 1 g P/kg feed.

2 Without mineral P supplementation.

3 LB = Lohmann Brown-Classic.

4 LSL = Lohmann LSL-Classic.

a–d Different superscript letters within a column and means of two-way-interaction and indicate significant interaction effects among two factors (< 0.05).

The expression of some genes related to intracellular InsP metabolism in the jejunum (*PRKCA, PIP4K2B, PI4K2B, PLCG1, SACM1L, PIK3C3, INPP5J*) was higher in LB than in LSL hens (Table 8; *P* ≤ 0.040). The expression of *SACM1L* and *ITPK1* was higher in wk 24 than in wk 19 (*P* ≤ 0.032) and the expression of *PIP4K2A* and *INPP5D* was higher in wk 19 than in wk 24 (*P* ≤ 0.011). The gene *PIP5K1B* was higher expressed in wk 24 P- than in wk 19 P- (period × P: *P* = 0.019). The gene *ITPK1* was higher expressed in wk 19 P+ than in other treatments (period × P: *P* = 0.008).

**Table 8:** Effect of production period, dietary P level, and hen strain on expression of genes of inositol phosphate metabolism in jejunum tissue.

Period	Wk 19				Week 24					*P* values						
P level	P+^1^		P-2		P+		P-			Period	Strain	P	P × Strain	Period × Strain	Period × P	Period × P × Strain
Strain	LB^3^	LSL^4^	LB	LSL	LB	LSL	LB	LSL	SEM							
*PRKCA*	0.0015	0.0014	0.0018	0.0010	0.0023	0.0013	0.0017	0.0010	0.00027	0.468	0.001	0.211	0.529	0.326	0.371	0.208
*PIP4K2B*	0.0234	0.0199	0.0260	0.0167	0.0301	0.0200	0.0387	0.0211	0.00463	0.071	0.003	0.481	0.305	0.257	0.429	0.900
*PI4K2B*	0.0215	0.0189	0.0226	0.0150	0.0277	0.0192	0.0340	0.0183	0.00405	0.069	0.004	0.829	0.295	0.223	0.478	0.851
*PLCG1*	0.0006	0.0003	0.0005	0.0003	0.0004	0.0003	0.0004	0.0001	0.00010	0.121	0.009	0.467	0.594	0.594	0.998	0.297
*SACM1L*	0.0101	0.0092	0.0113	0.0077	0.0126	0.0102	0.0124	0.0103	0.00131	0.032	0.039	0.904	0.447	0.981	0.927	0.384
*PIK3C3*	0.0029	0.0025	0.0027	0.0021	0.0030	0.0026	0.0039	0.0025	0.00046	0.125	0.021	0.826	0.260	0.472	0.279	0.541
*INPP5J*	0.0048	0.0046	0.0070	0.0040	0.0055	0.0041	0.0050	0.0042	0.00092	0.495	0.040	0.640	0.401	0.679	0.459	0.186
*PIP5K1B*	0.0016	0.0007	0.0009	0.0007	0.0011	0.0007	0.0015	0.0012	0.00024	0.399	0.052	0.662	0.202	0.509	0.019	0.282
*PIP4K2A*	0.0048	0.0061	0.0038	0.0053	0.0029	0.0041	0.0045	0.0036	0.00058	0.002	0.083	0.625	0.236	0.101	0.062	0.141
*IMPAD1*	0.0011	0.0027	0.0016	0.0020	0.0020	0.0018	0.0019	0.0019	0.00044	0.931	0.149	0.837	0.395	0.075	0.986	0.265
*PIP5K1C*	0.0021	0.0021	0.0027	0.0024	0.0022	0.0017	0.0021	0.0013	0.00038	0.060	0.180	0.620	0.611	0.334	0.225	0.961
*ITPK1*	0.0135	0.0119	0.0097	0.0076	0.0083	0.0067	0.0094	0.0088	0.00146	0.026	0.164	0.254	0.897	0.732	0.008	0.730
*IMPA1*	0.0041	0.0044	0.0052	0.0040	0.0051	0.0052	0.0059	0.0046	0.00074	0.150	0.318	0.630	0.172	0.826	0.818	0.972
*INPP5D*	0.0015	0.0014	0.0014	0.0017	0.0011	0.0008	0.0008	0.0015	0.00024	0.011	0.422	0.402	0.070	0.695	0.745	0.366
*PLCD1*	0.0009	0.0008	0.0008	0.0007	0.0006	0.0010	0.0008	0.0009	0.00019	0.947	0.483	0.862	0.690	0.143	0.665	0.669
*MTMR6*	0.0019	0.0020	0.0019	0.0019	0.0024	0.0031	0.0041	0.0026	0.00075	0.022	0.730	0.560	0.225	0.644	0.463	0.282

*PRKCA*, protein kinase C alpha; *PIP4K2B*, phosphatidylinositol-5-phosphate 4-kinase type 2 beta; *PI4K2B*, phosphatidylinositol 4-kinase type 2 beta; *PLCG1*, phospholipase C gamma 1; *SACM1L*, SAC1 suppressor of actin mutations 1-like (yeast); *PIK3C3*, phosphatidylinositol 3-kinase catalytic subunit type 3; *INPP5J*, inositol polyphosphate-5-phosphatase J; *PIP5K1B*, phosphatidylinositol-4-phosphate 5-kinase type 1 beta; *PIP4K2A*, phosphatidylinositol-5-phosphate 4-kinase type 2 alpha; *IMPAD1*, inositol monophosphatase domain containing 1; *PIP5K1C*, phosphatidylinositol-4-phosphate 5-kinase; type I; gamma; *ITPK1* = inositol-tetrakisphosphate 1-kinase; *IMPA1*, inositol monophosphatase 1; *INPP5D*, inositol polyphosphate-5-phosphatase D; *PLCD1*, phospholipase C delta 1; *MTMR6*, myotubularin related protein 6

1 supplemented with 1 g P/kg feed.

2 without mineral P supplementation.

3 LB = Lohmann Brown-Classic.

4 LSL = Lohmann LSL-Classic.

## DISCUSSION

Recommendations for NPP concentration of feed for laying hens, assuming 110 g ADFI, are 2.3 g/kg (National Research Council, 1994), 2.9 to 3.6 g/kg (Gesellschaft für Ernährungsphysiologie, 1999), 3.7 to 3.8 g/kg (Rostagno et al., 2017) and 3.8 g/kg (“available P”; Lohmann Breeders, 2022) and thus close to or higher than the actual P requirement (Rodehutscord et al., 2023). As we have seen no major effects on performance and metabolic traits when reducing dietary NPP from 2.5 g/kg by 20% (Sommerfeld et al., 2020b), we chose to consider the complete renunciation of mineral P in the design of this study. Further, we aimed to test the influence of the physiological status of the hens when using the same diet in the periods before the onset of egg laying (wk 19) and after it (wk 24). By adapting the light regime, we tried to ensure the hens in the first period remained without eggs. By shortening the prelayer feeding phase, we ensured that hens of both periods received the same layer feed during the sampling periods. As intended, no hens laid eggs until the end of the first period in wk 19. In the second period, 7.5% of the hens laid no egg at all, 15% laid their first egg in wk 21, 47.5% laid their first egg in wk 22, and 30% laid their first egg in wk 23 (data not shown). Thus, upon sampling in wk 24, almost all hens had started laying activity.

### Effects of Mineral Phosphorus Renunciation on Phytate Degradation

The results confirmed the first hypothesis that renunciation of mineral P increases endogenous phytate degradation. Concentrations of InsP_6_, Ins(1,2,4,5,6)P_5_, and Ins(1,2,3,4,5)P_5_ in duodenum+jejunum were lower with P- than P+ and lower in wk 24 than wk 19. As a higher P and Ca concentration in the intestine was found in wk 19 than wk 24, this suggests an interaction of these minerals with InsP_6_ and leading to inhibition of the initial InsP_6_ degradation step. Potential mechanisms behind it are end product inhibition of the endogenous phosphatases (McCuaig et al., 1972), a change of the intestinal pH, or the complexation of InsP_6_, making it less accessible for phosphatases (Angel et al., 2002). This aligns with the correlation between duodenum+jejunum InsP_6_ and duodenum+jejunum P concentrations in wk 19 (*r* = 0.627; *P* < 0.001), but not wk 24. The correlation between ileum InsP_6_ and ileum P concentrations (*r* = 0.812; *P* < 0.001) across both periods further indicates a diminishing effect of supplemented P on the initial InsP_6_ degradation step. This is consistent with data from broiler studies summarized by Rodehutscord et al. (2022) that showed a reduction in endogenous InsP_6_ degradation by 1.6 g with each gram of supplemented mineral P. Such an effect of mineral P on InsP_6_ degradation might be explainable by a decreased activity of endogenous phosphatases due to P oversupply (McCuaig et al., 1972). InsP isomers lower phosphorylated than InsP_5_ were not detected and together with the lack of a P supply effect on intestinal MI concentrations, this suggests no further degradation occurred. However, in a previous laying hen experiment (Sommerfeld et al., 2020b), 2 InsP_4_ and one InsP_3_ isomers were found in the ileum. As mineral P was present in all diets of that study, even in the P-reduced diet, this suggests that unknown factors promoted a faster degradation of lower InsP isomers in the present study compared to that by Sommerfeld et al. (2020b).

### Effects of the Transition From Pullet to Laying Hen

The intestinal release of MI from phytate is affected by diet composition (Sommerfeld et al., 2018; Novotny et al., 2023a,b). However, the inhibiting effect of P supplementation on InsP_6_ concentration in duodenum+jejunum of hens was not present throughout the whole phytate degradation cascade. The concentration of MI in duodenum+jejunum was only affected by the production period and was higher in wk 24 than wk 19. This matches the lower InsP_6_ concentration in wk 24 than in wk 19. Possibly, this was less of an effect of the period or age as rather an effect of the higher Ca concentration in the duodenum+jejunum in wk 19 than wk 24 despite equal Ca concentration in the feed. This would confirm our third hypothesis that metabolic responses exist between hens in wk 19 and wk 24 of age and this is explainable by the higher requirement of Ca at the beginning of laying activity, suggesting an increased intestinal absorption in this period. Decreased activity of mucosal phosphatases inhibited by Ca has been shown before (McCuaig et al., 1972; Applegate et al., 2003). This might be either caused by phytate-Ca complexation with reduction mainly on the initial step of phytate dephosphorylation or by direct inhibition of the mucosal phosphatases, reducing their activity (Brun et al., 2006) and inhibiting dephosphorylation of the lower InsP isomers. The Ca concentration in the digesta of duodenum+jejunum differed by 16 g/kg DM between wk 19 and 24 and might, therefore, have had a greater impact on the degradation of InsP isomers than the laying hen strain or dietary P level. *Myo*-inositol in jejunum and ileum was solely negatively affected by the dietary Ca concentration in the study by Sommerfeld et al. (2020b) and not by any other factors, strengthening the assumption mentioned above that the endogenous phosphatases responsible for the degradation of the less phosphorylated InsP to MI are more responsive to dietary Ca than P. A correlation between Ca and MI concentration in duodenum+jejunum (*r* = -0.604; *P* < 0.001) also suggests MI release-inhibiting function of Ca. The findings of this experiment suggest that most of the traits measured were mainly affected by the Ca and P concentration in the small intestine that occurred although the Ca content of the feed was unchanged.

### Effects on Jejunal Intracellular Metabolism

*Myo*-inositol is an important compound of membranes and signaling pathways in cells (Huber, 2016). Therefore, a higher release of MI from InsP during the gastrointestinal passage with a possibly higher MI uptake by the enterocytes might affect cell membrane building and signaling cascades by up or down regulating relevant genes. Some of the genes involved in intracellular InsP metabolism were higher expressed in LB than LSL hens and differences existed in the InsP and MI concentrations in the intestine between LB and LSL hens. The jejunal gene expression pattern of transcripts associated with intracellular MI metabolism reflects interactions between intestinal digesta composition and intracellular signaling cascades. Therefore, the fourth hypothesis seems partly to be confirmed, whereby the dietary P level did not cause the changes in intestinal InsP concentrations, as we hypothesized, but the hen strain did. Whether the upregulation of these genes is beneficial for the LB hen metabolism cannot be assessed at this time.

### Effects on Intestinal Phosphorus and Calcium Metabolism, Blood Concentrations, and Retention

The P concentrations in duodenum+jejunum were higher in wk 19 when hens were fed P+. In the ileum, it was generally higher in wk 19 than wk 24 and higher when P+ was fed. In these treatments, the intestinal concentrations of Ca were also elevated, suggesting the presence of Ca-P complexes. It is known that Ca can increase the P flow in the ileum and thus the P excretion (Rodehutscord et al., 2002) by depressing P absorption (Hurwitz and Bar, 1965). Phosphorus retention was low overall and increased by mineral P supplementation in the present study. In the study by Jing et al. (2018), P retention was also at or below 15% but was not affected by P supplementation. In a study of Rodehutscord et al. (2002), P retention of laying hens was at or below 13% but only affected by Ca supplementation. Possibly, the slightly higher P concentrations in the diets of Jing et al. (2018) and Rodehutscord et al. (2002) might explain these differences.

The second hypothesis that the 2 laying hen strains react differently to the dietary P reduction has to be partly rejected as the interaction between the hen strain and dietary P level was not significant in most cases. However, P retention was one of the few traits affected by an interaction of strain with dietary P level. Hens of the LB strain fed P- had a much lower P retention than LB hens fed P+ or LSL hens. As the amount of retained P was only affected by the dietary P level, this might indicate a slightly more efficient utilization of P by LSL hens when dietary P is not in excess.

Due to higher feed intake in wk 24, P intake was higher than in wk 19 and higher for P+ than P- diets. However, the higher P intake due to higher feed intake led to a higher P excretion, so the period did not affect P retention. In contrast, a higher dietary P level led to a higher P retention, suggesting a higher or more efficient utilization of the mineral P supplement. The higher P retention due to higher dietary P was reflected in a higher plasma P concentration. As the renunciation of P did not decrease performance, one might suggest that these hens had no disadvantage of the reduced P despite a lowered plasma P level. Nevertheless, this experiment was not designed to study P requirement and thus, the performance data should not be overemphasized due to the relatively short observation period. Further, indicators of bone mineralization, such as bone ash, have not yet been measured. As the laying hen prioritizes egg production, a P deficiency might become visible in bone demineralization before becoming visible in a performance decrease (Pongmanee et al., 2020).

Calcium intake during sampling was higher in wk 24 than 19 due to higher feed intake. However, the amount of excreted Ca did not differ between periods, resulting in a higher amount of retained Ca in wk 24, which is explainable by a higher need for egg production in this period. Calcium retention was highly correlated with the number of eggs laid since laying commenced (r = 0.736, *P* < 0.001) and the produced egg mass during excreta collection (r = 0.798, *P* < 0.001). This confirms how much the hen relies on a higher Ca availability with increasing egg production. Before the onset of egg laying, plasma total Ca was at an overall lower level despite the same Ca concentration in the feed. The plasma Ca pool increases when hens start laying eggs to ensure the high Ca flow needed for eggshell calcification, besides the need for Ca for metabolic processes and bone mineralization. However, the plasma Ca pool is not only supplied by the absorbed Ca but also by Ca mobilized from the medullary bone, which occurs mainly in the dark period without Ca intake (Whitehead, 2004; Sinclair-Black et al., 2023). This demineralization of the bone also causes P mobilization, which exemplifies the strong interrelationship between P and Ca in the intestine, blood, and bone. The concentrations of P and Ca in the blood plasma of laying hens follow a circadian rhythm with peaks during the egg shell formation when Ca is needed most (Frost and Roland, 1991). In the present study, plasma samples were taken once from each hen and at intervals of 15 min between individual hens starting at 9 o'clock in the morning. For the exact interpretation of plasma metabolites relevant to egg formation, the time of oviposition of each individual hen should have been known. We did not record the exact time of oviposition of each hen. However, most of the hens laid their egg during a short time span in the morning (before 9 o'clock), resulting in a similar circadian pattern. As we implemented the factor block, consisting of 4 metabolic units in the sampling sequence, in the statistical model, the course over time was considered in the results. However, the block effect was not significant for plasma P and Ca, which is why we assume no great diurnal effect, at least in the period of sampling.

Plasma Ca was decreased by dietary P supplementation in wk 19. In contrast, after the onset of egg laying, the Ca concentration was increased by P supplementation. This is also reflected in the correlations between plasma P and plasma Ca that were *r* = -0.699, *P* < 0.001 in wk 19 and *r* = 0.651, *P* < 0.001 in wk 24. It was also consistent with the Ca concentrations in duodenum+jejunum, being on a higher level in wk 19 than 24 and numerically higher in P+ than P- in wk 19 and vice versa in wk 24. This suggests strong connections between P and Ca absorption (Paul and Snetsinger, 1969; Reichmann and Connor, 1977). Despite more dietary P reaching the small intestine in wk 24, the P concentration in duodenum+jejunum did not differ between treatments, but plasma P was increased. Simultaneously, Ca concentration in duodenum+jejunum decreased and increased in plasma with P+. This suggests that with more Ca, more P was absorbed and thus present in the blood. Possibly, the higher Ca need of the hens in wk 24 increased the absorption of Ca from duodenum+jejunum and when more P was offered by the diet, more of both elements could be absorbed. In wk 19, more P was found in duodenum+jejunum with P+ and simultaneously more Ca, resulting in a lower plasma Ca concentration. In this period prior to laying activity, when the Ca need is not that high yet and Ca absorption is on a lower level, the formation of Ca-P complexes in the small intestine (Paul and Snetsinger, 1969) might play a larger role, resulting in the aforementioned observations.

## CONCLUSIONS

The renunciation of mineral P seemed to have provoked mechanisms that increased endogenous phytate degradation in laying hens without translating into a reduced P excretion. This suggests that phytate degradation in laying hens exists and is manipulable by diet composition, but it might not be relevant for the P supply of the hen. Differences in metabolic responses between the 2 production periods before and after the onset of egg laying (wk 19 and 24) existed. This seemed to be attributable to the higher Ca concentration in intestinal digesta in wk 19 compared to wk 24 caused by higher Ca needs for egg production. Although the laying hen strains differed in some response traits, they did not react differently to the dietary P levels. However, there was an indication for a slightly more efficient P utilization by LSL hens when dietary P was not in excess. The relevance of these strain differences for practical implications still needs to be elucidated.

## DISCLOSURES

MFö received speaker fees from Kyowa Kirin without relevance to this study. The authors declare they have no conflict of interest.

## References

[bib0001] Ahmadi H., Rodehutscord M. (2012). A meta-analysis of responses to dietary nonphytate phosphorus and phytase in laying hens. Poult. Sci..

[bib0002] Angel R., Tamim N.M., Applegate T.J., Dhandu A.S., Ellestad L.E. (2002). Phytic acid chemistry: Influence on phytin-phosphorus availability and phytase efficacy. J. Appl. Poult. Res..

[bib0003] Applegate T.J., Angel R., Classen H.L. (2003). Effect of dietary calcium, 25-hydroxycholecalciferol, or bird strain on small intestinal phytase activity in broiler chickens. Poult. Sci..

[bib0004] Boguhn J., Baumgärtel T., Dieckmann A., Rodehutscord M. (2009). Determination of titanium dioxide supplements in different matrices using two methods involving photometer and inductively coupled plasma optical emission spectrometer measurements. Arch. Anim. Nutr..

[bib0005] Brun L.R.M., Brance M.L., Rigalli A., Puche R.C. (2006). Effect of calcium on rat intestinal alkaline phosphatase activity and molecular aggregation. J. Enzyme Inhib. Med. Chem..

[bib0006] Frost T.J., Roland S.R. (1991). Effects of dietary phosphorus levels on circadian patterns of plasma 1,25-dihydroxycholecalciferol, total calcium, ionized calcium and phosphorus in laying hens. Poult. Sci..

[bib0007] Gesellschaft für Ernährungsphysiologie (1999).

[bib0008] Gonzalez-Uarquin F., Sommerfeld V., Rodehutscord M., Huber K. (2021). Interrelationship of *myo*-inositol pathways with systemic metabolic conditions in two strains of high-performance laying hens during their productive life span. Sci. Rep..

[bib0009] Huber K., Walk C.L., Kühn I., Stein H.H., Kidd M.T., Rodehutscord M. (2016). Pages 53-60 in Phytate Destruction - Consequences for Precision Animal Nutrition.

[bib0010] Hurwitz S., Bar A. (1965). Absorption of calcium and phosphorus along the gastrointestinal tract of the laying fowl as influenced by dietary calcium and egg shell formation. J. Nutr..

[bib0011] Jing M., Zhao S., Rogiewicz A., Slominski B.A., House J.D. (2018). Assessment of the minimal available phosphorus needs of laying hens: implications for phosphorus management strategies. Poult. Sci..

[bib0012] Lohmann B. (2022). Lohmann Brown-Classic Management Guide.

[bib0013] McCuaig L.W., Davies M.I., Motzok I. (1972). Intestinal alkaline phosphatase and phytase of chicks: Effect of dietary magnesium, calcium, phosphorus and thyroactive casein. Poult. Sci..

[bib0014] Novotny M., Sommerfeld V., Krieg J., Kühn I., Huber K., Rodehutscord M. (2023). Comparison of mucosal phosphatase activity, phytate degradation, and nutrient digestibility in 3-week-old turkeys and broilers at different dietary levels of phosphorus and phytase. Poult. Sci..

[bib0015] Novotny M., Sommerfeld V., Krieg J., Kühn I., Huber K., Rodehutscord M. (2023). Mucosal phosphatase activity, phytate degradation, and mineral digestibility in 6-week-old turkeys and broilers at different dietary levels of phosphorus and phytase and comparison with 3-week-old animals. Poult. Sci..

[bib0016] National Research Council (1994). Nutrient requirements of domestic animals. Nutrient Requirements of Poultry.

[bib0017] Omotoso A.O., Reyer H., Oster M., Ponsuksili S., Trakooljul N., Muráni E., Sommerfeld V., Rodehutscord M., Wimmers K. (2021). Jejunal transcriptomic profiling of two layer strains throughout the entire production period. Sci. Rep..

[bib0018] Paul H.S., Snetsinger D.C. (1969). Dietary calcium and phosphorus and variations in plasma alkaline phosphatase activity in relationship to physical characteristics of egg shells. Poult. Sci..

[bib0019] Pongmanee K., Kühn I., Korver D.R. (2020). Effects of phytase supplementation on eggshell and bone quality, and phosphorus and calcium digestibility in laying hens from 25 to 37 wk of age. Poult. Sci..

[bib0020] Reichmann K.G., Connor J.K. (1977). Influence of dietary calcium and phosphorus on metabolism and production in laying hens. Br. Poult. Sci..

[bib0021] Reyer H.M.O., Ponsuksili S., Trakooljul N., Omotoso A.O., Iqbal M.A., Muráni E., Sommerfeld V., Rodehutscord M., Wimmers K. (2021). Transcriptional responses in jejunum of two layer chicken strains following variations in dietary calcium and phosphorus levels. BMC Genomics.

[bib0022] Rodehutscord M., Sanver F., Timmler R. (2002). Comparative study on the effect of variable phosphorus intake at two different calcium levels on P excretion and P flow at the terminal ileum of laying hens. Arch. Anim. Nutr..

[bib0023] Rodehutscord M., Sommerfeld V., Kühn I., Bedford M.R., Bedford M.R., Partridge G., Hruby M., Walk C.L. (2022). Pages 124-152 in Enzymes in Farm Animal Nutrition.

[bib0024] Rodehutscord M., Sommerfeld V., Angel C.R., Korver D.R. (2023). Minimum phosphorus requirements for laying hen feed formulations. Poult. Sci..

[bib0025] Rostagno H.S., Albino L.F.T., Hannas M.I., Donzele J.L., Sakomura N.K., Perazzo F.G., Saraiva A., de Abreu M.L.T., Rodrigues P.B., de Oliveira R.F., de Toledo Barreto S.L., Brito C.O., Rostagno H.S. (2017). Departamento de Zootecnia.

[bib0026] Sinclair-Black M., Garcia R.A., Ellestad L.E. (2023). Physiological regulation of calcium and phosphorus utilization in laying hens. Front. Physiol..

[bib0027] Sommerfeld V., Schollenberger M., Kühn I., Rodehutscord M. (2018). Interactive effects of phosphorus, calcium, and phytase supplements on products of phytate degradation in the digestive tract of broiler chickens. Poult. Sci..

[bib0028] Sommerfeld V., Huber K., Bennewitz J., Camarinha-Silva A., Hasselmann M., Ponsuksili S., Seifert J., Stefanski V., Wimmers K., Rodehutscord M. (2020). Phytate degradation, myo-inositol release, and utilization of phosphorus and calcium by two strains of laying hens in five production periods. Poult. Sci..

[bib0029] Sommerfeld V., Omotoso A.O., Oster M., Reyer H., Camarinha-Silva A., Hasselmann M., Huber K., Ponsuksili S., Seifert J., Stefanski V., Wimmers K., Rodehutscord M. (2020). Phytate degradation, transcellular mineral transporters, and mineral utilization by two strains of laying hens as affected by dietary phosphorus and calcium. Animals.

[bib0030] Verband Deutscher Landwirtschaftlicher Untersuchungs- und Forschungsanstalten (VDLUFA) (2007).

[bib0031] Whitehead C.C. (2004). Overview of bone biology in the egg-laying hen. Poult. Sci..

[bib0032] Zeller E., Schollenberger M., Kühn I., Rodehutscord M. (2015). Hydrolysis of phytate and formation of inositol phosphate isomers without or with supplemented phytases in different segments of the digestive tract of broilers. J. Nutr. Sci..

